# Patient-specific template and electromagnetic navigation assisted bilateral periacetabular osteotomy for staged correction of bilateral injury-induced hip dysplasia: a case report

**DOI:** 10.1093/jhps/hnab054

**Published:** 2021-08-24

**Authors:** Peter Brumat, Rene Mihalič, Črt Benulič, Anže Kristan, Rihard Trebše

**Affiliations:** Valdoltra Orthopaedic Hospital, Jadranska cesta 31, Ankaran 6280, Slovenia; Faculty of Medicine, University of Ljubljana, Vrazov trg 2, Ljubljana 1000, Slovenia; Valdoltra Orthopaedic Hospital, Jadranska cesta 31, Ankaran 6280, Slovenia; Faculty of Medicine, University of Ljubljana, Vrazov trg 2, Ljubljana 1000, Slovenia; Faculty of Medicine, University of Ljubljana, Vrazov trg 2, Ljubljana 1000, Slovenia; Department of Traumatology, Division of Surgery, University Medical Centre Ljubljana, Ljubljana, Zaloška 7, Ljubljana 1000, Slovenia; Faculty of Medicine, University of Ljubljana, Vrazov trg 2, Ljubljana 1000, Slovenia; Department of Traumatology, Division of Surgery, University Medical Centre Ljubljana, Ljubljana, Zaloška 7, Ljubljana 1000, Slovenia; Valdoltra Orthopaedic Hospital, Jadranska cesta 31, Ankaran 6280, Slovenia; Faculty of Medicine, University of Ljubljana, Vrazov trg 2, Ljubljana 1000, Slovenia

## Abstract

Periacetabular osteotomy (PAO) for pelvic fracture sequelae presents a challenge in hip preservation surgery due to a combination of complex conditions involving post-traumatic altered anatomy and technically demanding procedure, with high surgical risk involved. To address these challenging conditions and evade potential devastating complications, a combination of patient-specific template (PST) and electromagnetic navigation (EMN) guidance can be used to increase the safety of the procedure and the accuracy of the acetabular reorientation. Herein we report our experience utilizing a combined PST- and EMN-assisted bilateral PAO for staged correction of bilateral severe, injury-induced hip dysplasia. The presented case report describes a unique method of successful surgical treatment of severe, bilateral injury-induced hip dysplasia with combined 3-D printing technology (PST) and intra-operative electromagnetic computer-assisted navigation (EMN) aided technically demanding surgical procedure (PAO), which emphasizes the benefits of PST and EMN use in hip preservation surgery in patients with complex pathoanatomic circumstances.

## INTRODUCTION

Periacetabular osteotomy (PAO) enables the restoration of any type of acetabular orientation pathology, to regain a proper joint function, alleviate pain and prevent or delay osteoarthritis onset [1, 2]. Despite its widespread use, this type of osteotomy still is a technically demanding procedure with a protracted learning curve and a complication rate ranging from 6 to 37% [3]. Managing patients with severe pelvic fractures is one of the most challenging aspects of trauma care [4]. The surgical management aims to achieve hemostasis and provide mechanical stability of the pelvic ring as well as bony anatomy [5]. It is, however, not always possible to achieve all of the goals in the acute setting, and secondary reconstructions are sometimes needed. The reconstruction of pelvic fracture sequelae is complex and presents a challenge for the surgeon due to the technical difficulty and high surgical risk involved [6]. To address these challenging conditions and evade potential devastating complications, a combination of patient-specific template (PST) and electromagnetic navigation (EMN) guidance can be used to increase the safety of the procedure and the accuracy of the acetabular reorientation [7]. Herein we report our experience utilizing a combined PST- and EMN-assisted bilateral periacetabular osteotomy for staged correction of symptomatic bilateral severe, injury-induced hip dysplasia.

## CASE DESCRIPTION

**Fig. 1.**
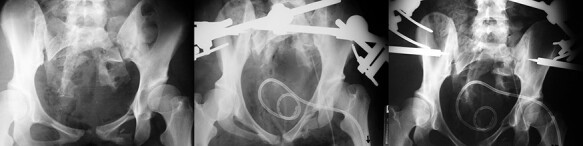
At the age of 20, patient was involved in a motor vehicle-pedestrian accident, sustaining a comminuted fracture of the sacrum and bilateral superior and inferior complicated fracture of the pubis with Morel-Lavallee lesion. X-rays of primary injury management (middle and right).

A 33-year-old female was referred to our hospital for treatment of chronic bilateral hip pain due to injury-induced severe hip dysplasia. Her major complaint was pain increasing when standing and intensifying in the recent years. She also reported limping, with hips giving way. At the age of 20, she was involved in a motor vehicle-pedestrian accident, sustaining a comminuted fracture of the sacrum and bilateral superior and inferior complicated fracture of the pubis with Morel-Lavallee lesion (Fig. 1). Primary injury management, reconstructive procedures and partial hardware removal were performed at another institution. As polytrauma sequelae, she reported a history of latent hyperthyroidism, residual left-sided limited active plantar flexion with L3-L5 dermatome hypoesthesia, but no dysfunction of sphincters. Passive left hip range of motion (ROM) was 100° of flexion, 10° of internal and 20° of external rotation, and 25° of abduction. Passive right hip ROM was 110° of flexion, 50° of internal and 25° of external rotation and 50° of abduction. Flexion, abduction and external rotation (FABER) and flexion, adduction, internal rotation (FADIR) tests were positive bilaterally. Apart from skin scarring from the injury and injury-related surgeries, she had intact overlying skin coverage in the pelvic region. No other neurovascular deficiencies were observed. The basic patient’s laboratory findings were within the normal range. Preoperative Harris Hip Score (HHS) was 43 for the left side and 41 for the right side.

**Fig. 2.**
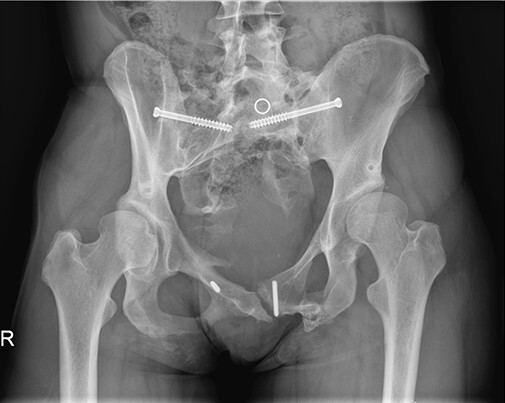
The imaging studies showed residual injury-induced alterations of the lumbosacral spine and pelvis with heavily altered bony anatomy.

The imaging studies showed residual injury-induced alterations of the lumbosacral spine and pelvis with heavily altered bony anatomy (Fig. 2). No definitive compression of the neural structures and no intra-articular hip abnormalities were observed on the imaging studies, including preserved hip cartilage and femoral head morphology bilaterally. The extent of the eventual bony abnormalities prior to the trauma was unknown.

**Fig. 3.**
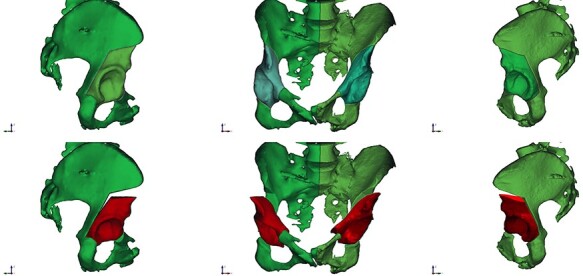
Patient personalized 3-D-based virtual pre-operative planning: 3D model of hips and pelvis is generated from the DICOM files obtained by the pre-operative CT scan of hips and pelvis. Lateral views (left and right) and anteroposterior view (middle).

**Fig. 4.**
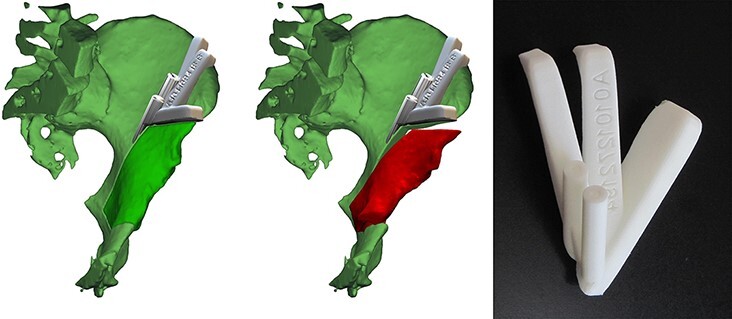
The design of the PST congruent to the exposed bone with two holes for guidance of the Kirschner wires along with the planned retroacetabular cut and a plane for the guidance of the supraacetabular cut for the left side PAO (left, middle). The PST made of biocompatible plastic (VisiJet, 3D systems, Rock Hill, SC, USA) for guiding the supra- and retro-acetabular osteotomy (right).

**Fig. 5.**
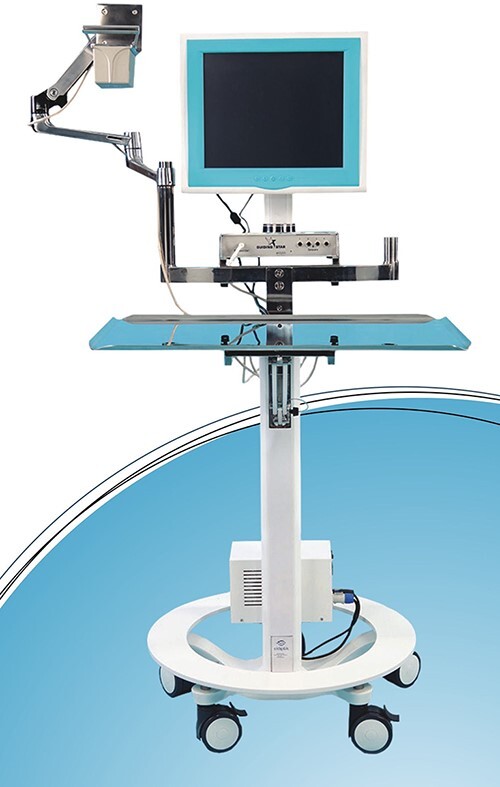
EMN system (Guiding Star, Ekliptik d.o.o., Ljubljana, Slovenia).

The patient was given a detailed explanation of her medical condition and treatment options. Based on history, clinical examination, and thorough diagnostics, we opted for the staged PST- and EMN-assisted bilateral PAO, using the modified Bernese PAO technique [8] and our patient personalized 3-D-based virtual pre-operative planning (Fig. 3), osteotomy (Fig. 4) and acetabular reorientation guidance (Fig. 5) concept [7]. For the pre-operative planning (Figs 3 and 4), we used the EBS medical software application (Ekliptik, d.o.o., Ljubljana, Slovenia). A DICOM format file from a pre-operative CT scan was uploaded into the EBS software where a virtual 3D model of the pelvis and both hips was generated (Fig. 3) [7]. The surgeon with the help of the software specialist then planned all four osteotomies and determined the desired acetabular fragment position and orientation for both sides on a 3D virtual pelvic model (Fig. 3), taking into consideration the virtual pre-operative hip ROM trial estimates to avoid any potential impingement and considering all three pelvic planes (Acetabular Index (AI), Lateral Center-Edge (LCE), Anterior Center-Edge (ACE) and acetabular version angle) [7]. We then designed the PST congruent to the exposed bone with two holes for guidance of the Kirschner wires along with the planned retroacetabular cut and a plane for the guidance of the supra-acetabular cut for the left and right side PAO separately (Fig. 4).

Both surgeries were performed by the last senior author (R.T.). We decided to operate on the left side first because we anticipated a faster recovery here. Cefazolin was used as antibiotic prophylaxis. The approach for the left side PAO was developed as described by Siebenrock *et al.* [8]. The osteotomies were performed with the help of PST for guiding the supra- and retro-acetabular osteotomy (Fig. 4) and EMN (Guiding Star, Ekliptik d.o.o., Ljubljana, Slovenia) for guiding the anterior ischial and superior pubic ramus osteotomy via navigated osteotome (Fig. 5), as described by Mihalič *et al.* [7]. The mobilized acetabular fragment was reoriented and medialized with the help of EMN (Fig. 5) [7] and secured with four 4,5 mm cortical screws. The intra-operative blood loss was ≅350 ml. The duration of surgery was 155 min. No drains were applied and unobstructed joint mobility was assured before wound closure.

**Fig. 6.**
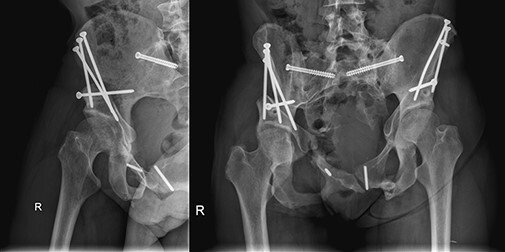
No clinical or radiological complications were seen on the regular follow-up visit 6 months after the second surgery. Axial view (left) and anteroposterior view (right).

The patient was dismissed 5 days after the surgery with tolerable pain. We administrated thromboprophylaxis for 35 days and a combination of oral non-opioid analgesics. The physiotherapy regimen was individually adjusted and supervised by an experienced physiotherapist. We allowed only partial (toe touch) weight bearing during the first 8 weeks. A similar surgical approach as described above for the left side PAO was also used for the right side PAO. The intra-operative blood loss was ≅500 ml and the duration of surgery was 175 min. No drains were applied and unobstructed joint mobility was assured before wound closure. The patient was dismissed on the sixth post-operative day with tolerable pain and unobstructed passive bilateral hip ROM. We followed the same post-operative protocol as for the first surgery. No clinical or radiological complications were seen on the regular follow-up visit 6 months after the second surgery (Fig. 6).

**Fig. 7.**
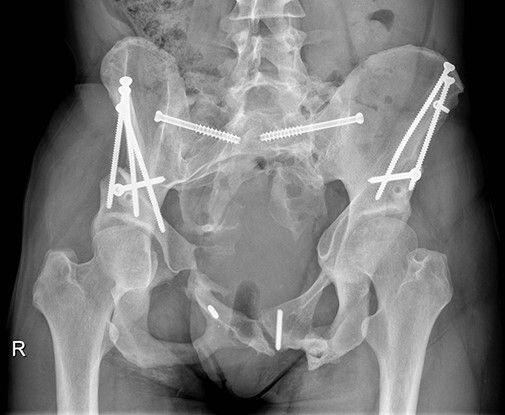
On the final in-office follow-up 30 months after the second surgery patient reported bilaterally undisturbed hip function and complete resolution of hip pain. Imaging showed correction according to the pre-operative plan.

On the final in-office follow-up 30 months after the second surgery patient reported bilaterally undisturbed hip function and complete resolution of hip pain. Imaging showed correction according to the pre-operative plan (Figs 3 and 7). The patient regained the ability to cope with the demands of daily life routine and recreational sports activity (Video 1). The passive left hip flexion was 100° with 10° of internal rotation, 20° of external rotation and 25° of abduction. The passive right hip flexion was 110° with 50° of internal rotation, 25° of external rotation and 25° of abduction. There were no signs of additional neurovascular deficiencies besides the previously identified residual left-sided limited active plantar flexion with L3-L5 dermatome hypoesthesia. We observed negative FABER and FADIR tests. The HHS was 91 for the left side and 84 for the right side.

## DISCUSSION

We present a unique case of a young woman who suffered severe pelvic trauma resulting in debilitating, bilateral, severe injury-induced hip dysplasia and consequential inability to cope with demands of daily life routine that improved significantly after bilateral PAO. Although the main indication for PAO presents symptomatic developmental hip dysplasia in young adults with preserved hip cartilage and no history of previous hip surgery or trauma, miscellaneous hip disorders can be addressed with PAO [2]. However, the surgical technique needed to perform PAO is technically demanding, with correct repositioning of the acetabulum the most important—and challenging—aspect of the procedure [9]. Given the complex circumstances of extreme anatomic alteration after an injury can, therefore, make this type of surgery technically even more demanding, particularly the execution of bony cuts and acetabular fragment correction. Particularly the right-side PAO in the presented case is interesting since it challenges the ability of the technique for AI correction on one side and displays the lower limit of the necessary contact between the acetabular fragment and posterior column that still allows for a successful and durable fusion. Moreover, residual tissue scarring after injury and injury-related surgeries, and ectopic bone with injury-induced altered anatomy make the visualization and recognition of landmarks not only difficult but also interfere with fragment mobilization and correction, which can sometimes be challenging even without complex conditions. The additional challenges in such conditions are surgical risks incurred by altered neurovascular anatomy with limited elasticity and mobility of these structures.

The benefits of computer assistance in hip preservation surgery, pelvic ring injury and acetabular fracture management are recognized [7, 10–12]. Three-dimensional printing may also come particularly valuable in complex cases. Its application may significantly reduce blood loss, operative time and fluoroscopy use [13]. To improve the safety and accuracy of the PAO in these complex circumstances presented by our patient, we implemented a combination of PST (Fig. 4) and EMN (Fig. 5) guidance [7]. Presented EMN system works on the principle of surface-based registration. Prior to the process of registration, the reference sensor is placed onto the iliac crest [7]. Several pelvic surface points are digitized with a probe and adjusted to the 3D model of hips and pelvis generated from the DICOM files obtained by the pre-operative CT scan of hips and pelvis [7]. The EMN system then gives the real-time feedback about the position of the osteotome and osteotomy planes [7]. The osteotomies are performed with the help of PST for guiding the supra- and retro-acetabular osteotomy (Fig. 4) and EMN for guiding the anterior ischial and superior pubic ramus osteotomy via navigated osteotome (Fig. 5) [7]. Before the osteotomized acetabular fragment is mobilized a tracker is firmly attached to it. While maneuvering the fragment one can intra-operatively see in the real-time on the screen the actual position of the osteotomized navigated fragment as well as the planned final position. When both match, the desired/planned position is achieved and the acetabular fragment can be fixed with the screws [7]. Our patient personalized 3-D based virtual pre-operative planning (Fig. 3), osteotomy (Fig. 4) and acetabular reorientation guidance (Fig. 5) concept thus allows for a safer and more accurate 3-D acetabular geometry restoration in all three pelvic planes regardless of the individual’s anatomic complexity, taking into account the AI, LCE, ACE and acetabular version angle while avoiding the under/over coverage and impingement pitfalls possibly incurred by the use of traditional fluoroscopy-guided freehand technique [7]. We believe that using a PST and/or EMN may be essential to reduce the potential harm to the minimum possible and to optimize the accuracy of the execution of the surgical plan, particularly in patients with complex pathoanatomic circumstances. However, it remains paramount to fully analyze and understand the given anatomical circumstances in complex conditions, to plan the surgery meticulously and to acknowledge potential benefits and complications, which can be devastating if disregarded. Since our patient was a young female, gender should also be taken into consideration when planning the surgery due to the potential impact of PAO on the delivery method [14].

The presented case report describes a unique method of successful surgical treatment of severe, bilateral injury-induced hip dysplasia with combined 3-D printing technology (PST) and intra-operative electromagnetic computer-assisted navigation (EMN) aided technically demanding surgical procedure (PAO), which emphasizes the benefits of PST and EMN use in hip preservation surgery in patients with complex pathoanatomic circumstances.

## Supplementary Material

hnab054_Supp

## Data Availability

The data underlying this article are available in the article and in its online supplementary material.
